# Clinical impact of complete atrioventricular block in patients with ST‐segment elevation myocardial infarction

**DOI:** 10.1002/clc.23510

**Published:** 2020-11-12

**Authors:** Yosuke Kawamura, Hiroaki Yokoyama, Kazutaka Kitayama, Naotake Miura, Misato Hamadate, Daiki Nagawa, Masashi Nozaka, Masamichi Nakata, Fumie Nishizaki, Kenji Hanada, Takashi Yokota, Masahiro Yamada, Takumi Higuma, Hirofumi Tomita

**Affiliations:** ^1^ Department of Cardiology and Nephrology Hirosaki University Graduate School of Medicine Hirosaki Japan; ^2^ Division of Cardiology, Department of Internal Medicine St. Marianna University School of Medicine Kawasaki Japan

**Keywords:** complete atrioventricular block, percutaneous coronary intervention, ST‐segment elevation myocardial infarction

## Abstract

Complete atrioventricular block (CAVB) is a common complication of ST‐segment elevation myocardial infarction (STEMI). Although STEMI patients complicated with CAVB had a higher mortality in the thrombolytic era, little is known about the impact of CAVB on STEMI patients who underwent primary percutaneous coronary intervention (PCI). The study aimed at evaluating the clinical impact of CAVB on STEMI patients in the primary PCI era. We consecutively enrolled 1295 STEMI patients undergoing primary PCI within 24 hours from onset. Patients were divided into two groups according to the infarct location: anterior STEMI (n = 640) and nonanterior STEMI (n = 655). The outcomes were all‐cause death and major adverse cardiocerebrovascular events (MACCE) with a median follow‐up period of 3.8 (1.7–6.6) years. Eighty‐one patients (6.3%) developed CAVB. The incidence of CAVB was lower in anterior STEMI patients than in nonanterior STEMI (1.7% vs 10.7%, *p* < .05). Anterior STEMI patients with CAVB had a higher incidence of all‐cause deaths (82% vs 20%, *p* < .05) and MACCE (82% vs 25%, *p* < .05) than those without CAVB. Although higher incidence of all‐cause deaths was found more in nonanterior STEMI patients with CAVB compared with those without CAVB (30% vs 18%, *p* < .05), there was no significant difference in the incidence of MACCE (24% vs 19%). Multivariate analysis showed that CAVB was an independent predictor for all‐cause mortality and MACCE in anterior STEMI patients, but not in nonanterior STEMI. CAVB is rare in anterior STEMI patients, but remains a poor prognostic complication even in the primary PCI era.

## 1 INTRODUCTION

Complete atrioventricular block (CAVB) is a common complication of acute myocardial infarction (AMI), and previous reports show that the incidence of CAVB is 3% to 11%.^1^, ^2^, ^3^, ^4^, ^5^, ^6^, ^7^, ^8^ CAVB can occur in ST‐segment elevation myocardial infarction (STEMI) patients. However, it is more common in inferior STEMI patients.^1^, ^2^, ^3^, ^4^, ^6^, ^7^, ^8^, ^9^, ^10^, ^11^, ^12^ Aplin et al. reported that STEMI patients with CAVB had a higher mortality irrespective of their infarct locations in the thrombolytic era.^2^, ^3^, ^4^, ^5^, ^6^, ^7^, ^8^, ^10^ This increased incidence was more prominent in the anterior than in the inferior infarct location. Randomized studies have demonstrated that percutaneous coronary intervention (PCI) is a more effective treatment strategy in patients with STEMI rather than thrombolytic therapy.^13^, ^14^, ^15^ However, little is known about the incidence of CAVB and its prognostic impact on STEMI patients treated with primary PCI. In this study, we aimed at examining the incidence, characteristics, and outcomes in STEMI patients complicated with CAVB in the era of primary PCI.

## 2 METHODS

### 2.1 Study population

This was a retrospective and observational study. We evaluated consecutive STEMI patients admitted in the Hirosaki University Hospital from January 2007 to December 2016 within 24 hours of symptom onset, and had underwent primary PCI. We defined STEMI as an elevated cardiac biomarker with two of the following^1^: typical chest pain lasting >20 minutes and^2^ ECG showing new ST‐segment elevation ≥2 mm in at least two contiguous precordial leads or ≥ 1 mm in at least two contiguous limb leads, or newly apparent left bundle branch block. We divided patients by evaluating lesions using emergent coronary angiography as follows: (a) anterior STEMI patients with culprit in left anterior descending artery (LAD) or left main trunk and (b) nonanterior STEMI patients with culprit in right coronary artery (RCA) or left circumflex artery (LCX). We defined CAVB as complete interruption of atrioventricular (AV) conduction with dissociation of P waves and QRS complexes and the existence of junctional or ventricular escape rhythms with a rate less than the atrial rate. In this study, we considered CAVB as follows: documented CAVB in continuous cardiac monitoring in ambulances, previous hospital, or at any point during the hospitalization whether it was temporary or sustained. The Ethics Committee of our Institution approved this study.

### 2.2 Data collection and follow‐up

We obtained baseline clinical characteristics including previous medical history, and analyzed laboratory data at admission with electrocardiography (ECG) before PCI and during hospitalization. We assessed the left ventricular ejection fraction (LVEF) at acute phase by transthoracic echocardiography or left ventriculography during hospitalization. In this phase, we evaluated the number of diseased vessels defined as more than 75% major coronary stenosis, and judged thrombolysis in myocardial infarction (TIMI) grade flow after emergent PCI. The primary outcome of this study was all‐cause death and the secondary outcome was major adverse cardiocerebrovascular events (MACCE), defined as a composite of cardiovascular death, nonfatal myocardial infarction, nonfatal stroke and hospitalization due to acute decompensated heart failure (ADHF). Follow‐up started on the day of admission and we followed up patients until 2018 with a median follow‐up period of 3.8 (1.7–6.6) years. We obtained follow‐up data by reviewing our hospital records, interviewing the patients or their families by telephone, or examining the outpatient clinic records.

### 2.3 Statistical analysis

We expressed continuous variables as mean ± standard deviation or median (interquartile range), and categorical variables as frequencies and percentages. We compared continuous variables using one‐way analysis of variance, and calculated the statistical significance of differences using the Tukey–Kramer test. We used the Mann–Whitney U test for nonparametric variables and the chi‐square analysis to compare categorical variables. We estimated primary and secondary outcomes using the Kaplan–Meier method and compared them using the log‐rank test. We performed univariate and multivariate analyses for the predictors of all‐cause death and MACCE using the Cox proportional hazards regression. The variables used for analysis included CAVB, age, and male gender (Model A), and CAVB, age, male gender, hypertension, dyslipidemia, diabetes mellitus, smoking, estimated glomerular filtration rate (eGFR) <60 mL/min/1.73 mL^2^, Killip classification ≥2, log peak creatine phosphokinase (CPK), LVEF <40%, and TIMI grade ≤ 1 (Model B). Hazard ratios (HRs) and 95% confidence intervals (CIs) were calculated. A *p* values less than .05 was considered as statistically significant. Statistical analyses were performed using JMP pro version 14 (SAS institute, Cary, NC).

## 3 RESULTS

### 3.1 Baseline characteristics

We consecutively enrolled and evaluated 1295 STEMI patients (1020 males; mean age, 66 ± 13 years). We diagnosed 640 patients (49%) with anterior STEMI, and 655 (51%) with nonanterior STEMI. The incidence of CAVB was significantly lower in anterior STEMI patients than in nonanterior STEMI patients (11/640 = 1.7% vs 70/655 = 10.7%, *p* < .05) (Supplementary Figure). Baseline characteristics of the study population are shown in Table 1. Patients with CAVB were older compared to those without CAVB, though not significant. There were no differences in gender, coronary risk factors, and the prevalence of previous cardiocerebrovascular disease between the two groups. Patients with CAVB had a significantly higher prevalence of Killip classification ≥2 at admission (64% vs 23% in anterior STEMI and 23% vs 10% in nonanterior STEMI, respectively, both *p* < .05) and higher peak CPK and CPK‐MB levels irrespective of STEMI location. The prevalence of final TIMI 3 after primary PCI was significantly lower in patients with CAVB compared with those without (27% vs 82% in anterior STEMI and 67% vs 82% in nonanterior STEMI, respectively, both *p* < .05). Anterior STEMI patients with CAVB had significantly lower LVEF compared with those without CAVB (35 ± 12% vs 43 ± 11%, *p* < .05), while nonanterior STEMI patients with CAVB had a significantly higher LVEF compared with those without CAVB (52 ± 12% vs 50 ± 10%, *p* < .05). There was a higher tendency of multi‐vessel disease in anterior STEMI patients with CAVB compared with those without CAVB (73% vs 46%, *p* = .08), whereas similar tendency between nonanterior STEMI patients with CAVB and without (53% vs 57%, *p* = .48). Decreased renal function were observed in STEMI patients with CAVB. Time to reperfusion was significantly shorter in all patients and nonanterior STEMI patients with CAVB than in those without CAVB, whereas no significant difference was found between anterior STEMI patients with CAVB and without. Direct transfer to emergency room by the emergency services without going through primary care physicians was significantly more frequent in all patients and nonanterior STEMI patients with CAVB compared with those without CAVB (17% vs 5% in all patients and in nonanterior STEMI patients, respectively, both *p* < .05), and difference was close to significance between anterior STEMI patients with CAVB and without (18% vs 4%, *p* = .09).

**TABLE 1:** Baseline characteristics of the patients divided by the presence of complete atrioventricular block

	All patients			Anterior STEMI			Nonanterior STEMI		
	CAVB (+)(n = 81)	CAVB (−)(n = 1214)	*p* value	CAVB (+)(n = 11)	CAVB (−)(n = 629)	*p* value	CAVB (+) (n = 70)	CAVB (−) (n = 585)	*p* value
Age, years	69 ± 14	66 ± 12	.05	73 ± 14	66 ± 13	.08	68 ± 14	66 ± 12	.14
Male, n (%)	63 (78)	957 (79)	.82	8 (73)	499 (79)	.71	55 (79)	458 (78)	.96
BMI, kg/m^2^	23.7 ± 3.7	24.2 ± 3.6	.24	23.0 ± 3.1	24.3 ± 3.6	.22	23.9 ± 3.8	24.1 ± 3.6	.58
Coronary risk factor									
Diabetes mellitus, n (%)	41 (51)	592 (49)	.75	5 (45)	316 (50)	.75	36 (51)	276 (47)	.50
Hypertension, n (%)	54 (67)	891 (73)	.19	8 (73)	465 (74)	1.00	46 (66)	426 (73)	.21
Dyslipidemia, n (%)	64 (79)	973 (80)	.80	7 (64)	508 (81)	.24	57 (81)	465 (79)	.70
Current smoking, n (%)	34 (42)	499 (41)	.89	2 (18)	240 (38)	.22	32 (46)	259 (44)	.84
Previous MI, n (%)	4 (5)	122 (10)	.13	1 (9)	54 (9)	1.00	3 (4)	68 (12)	.06
Previous CABG, n (%)	2 (2)	15 (1)	.29	0 (0)	2 (1)	1.00	2 (3)	13 (2)	.74
Previous stroke, n (%)	10 (12)	113 (9)	.37	2 (18)	56 (9)	.26	8 (11)	57 (10)	.66
Killip class ≥2, n (%)	23 (14)	201 (17)	<.05	7 (64)	145 (23)	<.05	16 (23)	56 (10)	<.05
LVEF, %	50 ± 13	46 ± 11	<.05	35 ± 12	43 ± 11	<.05	52 ± 12	50 ± 10	<.05
Multi‐vessel disease, n (%)	45 (56)	623 (51)	.46	8 (73)	288 (46)	.08	37 (53)	335 (57)	.48
Time to reperfusion, min	232 (161–342)	292 (197–461)	<.05	310 (152–494)	292 (196–448)	.75	228 (167–329)	294 (198–474)	<.05
Direct transfer to ER, n (%)	14 (17)	59 (5)	<.05	2 (18)	28 (4)	.09	12 (17)	31 (5)	<.05
Final TIMI flow 3, n (%)	50 (62)	997 (82)	<.05	3 (27)	515 (82)	<.05	47 (67)	482 (82)	<.05
Laboratory findings									
eGFR, mL/min/1.73m^2^	54 ± 26	71 ± 26	<.05	44 ± 25	72 ± 26	<.05	56 ± 26	70 ± 27	<.05
Peak CPK, IU/L	3177 (1714‐5378)	2391 (1066‐4437)	<.05	12 009 (7197‐14 286)	2948 (1151‐5540)	<.05	2618 (1381‐4352)	1987 (976–3522)	<.05
Peak CPK‐MB, IU/L	288 (161–521)	238 (106–450)	<.05	757 (605–1692)	292 (115–525)	<.05	275 (150–423)	207 (99–353)	<.05

*Note:* Data are presented as frequency (percentage) for categorical variables and as mean ± standard deviation or median (interquartile range) for continuous variables. STEMI indicates ST‐segment elevation myocardial infarction.

Abbreviations: BMI, body mass index; CAVB, complete atrioventricular block; CABG; coronary artery bypass grafting; CPK; creatine phosphokinase, CPK‐MB; creatine phosphokinase myocardial isoform; ER, emergency room; eGFR, estimated glomerular filtration rate; MI, myocardial infarction; LVEF, left ventricular ejection fraction; TIMI, thrombolysis in myocardial infarction.

### 3.2 Clinical outcomes in anterior STEMI

As shown in Table 2, anterior STEMI patients with CAVB had a higher prevalence of cardiogenic shock compared with those without CAVB (64% vs 9%, *p* < .05) and ADHF during hospitalization (100% vs 20%, *p* < .05). In‐hospital mortality of anterior STEMI patients with CAVB reached 55%, which was significantly worse compared with those without CAVB (7%). Not only short‐term but long‐term mortality were higher in anterior STEMI patients with CAVB compared to those without CAVB. Prevalence of overall devise implantation did not differ between anterior STEMI patients with CAVB and without.

**TABLE 2:** Clinical outcomes of the patients divided by the presence of complete atrioventricular block

	All patients			Anterior STEMI			Nonanterior STEMI		
	CAVB (+) (n = 81)	CAVB (−) (n = 1214)	p value	CAVB (+) (n = 11)	CAVB (−)(n = 629)	p value	CAVB (+) (n = 70)	CAVB (−) (n = 585)	p value
Complications during hospitalization									
Cardiogenic shock, n (%)	20 (24)	85 (7)	<0.05	7 (64)	57 (9)	<0.05	13 (19)	28 (5)	<0.05
Decompensated heart failure, n (%)	31 (38)	175 (14)	<0.05	11 (100)	128 (20)	<0.05	20 (29)	47 (8)	<0.05
Ventricular arrhythmias, n (%)	11 (14)	113 (9)	0.21	5 (45)	66 (10)	<0.05	6 (9)	47 (8)	0.88
Device implantation, n (%)	4 (5)	14 (1)	<0.05	1 (9)	7 (1)	0.13	3 (4)	7 (1)	0.08
PPM, n (%)	2 (2)	4 (1)	<0.05	1 (9)	1 (1)	<0.05	1 (1)	3 (1)	0.36
ICD, n (%)	1 (1)	9 (1)	0.48	0 (0)	5 (1)	1.00	1 (1)	4 (1)	0.43
CRT, n (%)	1 (1)	1 (1)	0.12	0 (0)	1 (1)	1.00	1 (1)	0 (0)	0.11
Short‐term outcomes									
In‐hospital mortality, n (%)	9 (11)	59 (5)	<0.05	6 (55)	42 (7)	<0.05	3 (4)	17 (3)	0.46
30‐day mortality, n (%)	8 (10)	59 (5)	0.05	5 (46)	41 (7)	<0.05	3 (4)	18 (3)	0.49
Long‐term outcomes									
All‐cause death, n (%)	30 (37)	233 (19)	<0.05	9 (82)	128 (20)	<0.05	21 (30)	105 (18)	<0.05
MACCE, n (%)	26 (32)	268 (22)	<0.05	9 (82)	159 (25)	<0.05	17 (24)	109 (19)	0.26
Cardiac death, n (%)	14 (18)	112 (9)	<0.05	8 (73)	74 (12)	<0.05	6 (9)	38 (7)	0.45
Nonfatal MI, n (%)	4 (5)	64 (5)	1.00	0 (0)	27 (4)	1.00	4 (6)	37 (6)	1.00
Nonfatal stroke, n (%)	5 (6)	71 (6)	0.90	0 (0)	34 (5)	1.00	5 (7)	37 (6)	0.80
Hospitalization due to HF, n (%)	6 (7)	88 (7)	0.96	2 (18)	59 (9)	0.28	4 (6)	29 (5)	0.77

*Note:* Data are presented as frequency (percentage) for categorical variables. STEMI indicates ST‐segment elevation myocardial infarction.

Abbreviations: CAVB, complete atrioventricular block; CRT, cardiac resynchronization therapy; HF, heart failure; ICD, implantable cardioverter defibrillator; MACCE, major adverse cardiocerebrovascular event; MI, myocardial infarction; PPM, permanent pacemaker.

The detail time courses and outcomes of 11 anterior STEMI patients with CAVB are summarized in Supplementary Table. We confirmed CAVB in three patients before PCI and the rest developed CAVB during or after PCI. There were eight patients (73%) with multi‐vessel disease. All patients underwent successful primary PCI, and had their final TIMI flow of at least 2. Six patients required temporary transvenous pacing due to hemodynamic instability. CAVB was transient in 10 patients. However, we implanted a permanent pacemaker (PPM) in one patient at 10 days after STEMI onset due to a new‐onset CAVB after PCI. Six patients (55%) died during the hospitalization, and the cause of deaths were myocardial pump failure or cardiogenic shock. On the other hand, CAVB disappeared in all survivors.

**FIGURE 1:**
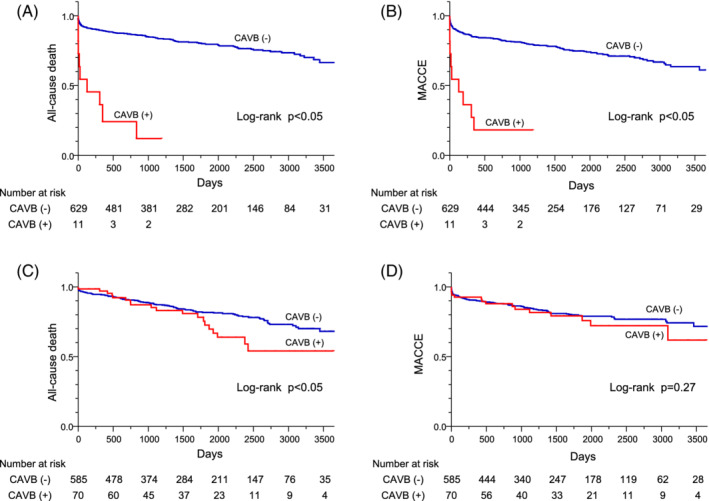
Comparisons of Kaplan–Meier curves for all‐cause death A, and for MACCE, B, in anterior STEMI patients with/without CAVB, and those for all‐cause death, C, and for MACCE, D, in nonanterior STEMI patients with/without CAVB. Cumulative incidence curves of STEMI patients with CAVB (red line) and without (blue line) are shown. MACCE; major adverse cardiocerebrovascular events, STEMI; ST‐segment elevation myocardial infarction, CAVB; complete atrioventricular block

Kaplan–Meier analyses also showed that all‐cause mortality and MACCE were significantly higher in anterior STEMI patients with CAVB compared to those without CAVB (*p* < .05 by Log‐rank test) (Figure 1(A) and (B)). Table 3 displays univariate and multivariate analyses for all‐cause mortality and MACCE in anterior STEMI patients. Univariate analysis showed that CAVB was significantly associated with all‐cause mortality (HR: 9.22; 95% CI: 4.30–17.38, *p* < .05) and MACCE (HR: 6.69; 95% CI: 3.15–12.47, *p* < .05), respectively. Multivariate Cox proportional hazard analysis showed that CAVB were associated with all‐cause mortality (HR: 3.01; 95% CI: 1.33–6.09, *p* < .05) and MACCE (HR: 2.23; 95% CI: 1.01–4.38, *p* < .05) in anterior STEMI patients, respectively. Furthermore, age, eGFR <60 mL/min/1.73 m^2^, Killip ≥2, and LVEF <40% were also independent predictors for both all‐cause mortality and MACCE. Meanwhile, the male gender was only for all‐cause mortality and log peak CPK was only for MACCE in anterior STEMI patients. These results indicate that anterior STEMI patients complicated with CAVB had poor outcomes even after successful primary PCI.

**TABLE 3:** Predictors of all‐cause death and MACCE in anterior STEMI patients

Factor	Univariate analysis			Multivariate analysis (Model A)			Multivariate analysis (Model B)		
	HR	95% CI	*p* value	HR	95% CI	*p* value	HR	95% CI	*p* value
All‐cause death									
CAVB	9.22	4.30–17.38	<.05	6.22	2.89–11.83	<.05	3.01	1.33–6.09	<.05
Age	1.07	1.05–1.09	<.05	1.07	1.05–1.09	<.05	1.06	1.04–1.08	<.05
Male gender	1.08	0.73–1.67	.70	1.47	0.98–2.28	.06	1.57	1.01–2.53	<.05
Hypertension	0.97	0.67–1.43	.88	−	−	−	0.93	0.61–1.43	.73
Dyslipidemia	0.55	0.38–0.81	<.05	−	−	−	0.71	0.48–1.08	.11
Diabetes mellitus	1.14	0.82–1.60	.44	−	−	−	1.22	0.85–1.76	.28
Smoking	0.56	0.37–0.81	<.05	−	−	−	1.07	0.67–1.68	.78
eGFR<60 mL/min/1.73mL^2^	5.31	3.77–7.55	<.05	−	−	−	2.57	1.73–3.83	<.05
Killip class ≥2	5.97	4.26–8.43	<.05	−	−	−	3.04	2.05–4.51	<.05
Log peak CPK	2.12	1.42–3.27	<.05	−	−	−	1.23	0.79–1.95	.36
LVEF <40%	2.99	2.12–4.25	<.05	−	−	−	1.70	1.15–2.52	<.05
Final TIMI flow ≤1	3.26	1.54–6.05	<.05	−	−	−	1.87	0.72–4.06	.18
MACCE									
CAVB	6.69	3.15–12.47	<.05	5.05	2.37–9.49	<.05	2.23	1.01–4.38	<.05
Age	1.05	1.03–1.06	<.05	1.00	1.03–1.06	<.05	1.04	1.02–1.06	<.05
Male gender	0.84	0.60–1.22	.35	1.05	0.74–1.52	.79	0.89	0.60–1.34	.58
Hypertension	0.98	0.70–1.39	.91	−	−	−	0.98	0.68–1.43	.90
Dyslipidemia	0.72	0.51–1.04	.08	−	−	−	0.91	0.62–1.34	.62
Diabetes mellitus	1.27	0.94–1.74	.12	−	−	−	1.27	0.92–1.77	.14
Smoking	0.75	0.54–1.03	.08	−	−	−	1.41	0.94–2.09	.09
eGFR<60 mL/min/1.73mL^2^	4.21	3.09–5.74	<.05	−	−	−	2.44	1.71–3.49	<.05
Killip class ≥2	4.55	3.35–6.17	<.05	−	−	−	2.16	1.50–3.10	<.05
Log peak CPK	2.37	1.64–3.49	<.05	−	−	−	1.51	1.00–2.28	<.05
LVEF <40%	2.95	2.16–4.04	<.05	−	−	−	1.76	1.24–2.49	<.05
Final TIMI flow ≤1	2.50	1.18–4.62	<.05	−	−	−	1.48	0.57–3.15	.38

Abbreviations: CI; confidence interval; HR indicates hazard ratio; Other abbreviations as in Tables 1, 2.

### 3.3 Clinical outcomes in nonanterior STEMI

Nonanterior STEMI patients with CAVB had a higher Killip classification than those without CAVB (Table 1). Cardiogenic shock and ADHF were significantly more in nonanterior STEMI patients with CAVB compared to those without CAVB (19% vs 5%, and 29% vs 8%, respectively, both *p* < .05) (Table 2). However, in‐hospital and 30‐day mortality did not differ between nonanterior STEMI patients with CAVB and those without CAVB. Although we found a significantly higher all‐cause mortality in nonanterior STEMI patients with CAVB compared to those without CAVB (30% vs 18%, *p* < .05), no significant difference was found in the incidence of MACCE between nonanterior STEMI patients with and without CAVB (24% vs 19%, *p* = .26) (Table 2). One nonanterior STEMI patient with CAVB regained sinus rhythm after PCI. However, PPM implantation was needed due to HV‐interval prolongation in electrophysiological study. We implanted implantable cardioverter defibrillator (ICD) or cardiac resynchronization therapy with defibrillator in two nonanterior STEMI patients with CAVB for primary or secondary prevention of sudden cardiac death. Among nonanterior STEMI patients without CAVB, seven patients needed permanent pacing devices; three underwent PPM because of SSS and four underwent ICD implantation for primary or secondary prevention of sudden cardiac death. Although Kaplan–Meier analyses showed a significantly higher incidence of all‐cause death in nonanterior STEMI patients with CAVB compared to those without CAVB (*p* < .05 by Log‐rank test), we observed no significant difference in the incidence of MACCE (*p* = .27) (Figure 1(C) and (D)). By multivariate analysis, CAVB was not associated with all‐cause mortality and MACCE in nonanterior STEMI patients (Table 4). Age, eGFR <60 mL/min/1.73 m^2^, Killip ≥2, and LVEF <40% were independently associated with both all‐cause mortality and MACCE in nonanterior STEMI patients, while diabetes mellitus was an independent predictor for MACCE.

**TABLE 4:** Predictors of all‐cause death and MACCE in nonanterior STEMI patients

Factor	Univariate analysis			Multivariate analysis (Model A)			Multivariate analysis (Model B)		
	HR	95% CI	*p* value	HR	95% CI	*p* value	HR	95% CI	*p* value
All‐cause death									
CAVB	1.76	1.07–2.75	<.05	1.48	0.90–2.33	.12	0.99	0.57–1.64	.98
Age	1.08	1.06–1.10	<.05	1.08	1.06–1.10	<.05	1.08	1.05–1.10	<.05
Male gender	0.78	0.52–1.19	.24	1.40	0.92–2.18	.12	1.35	0.86–2.18	.19
Hypertension	1.11	0.75–1.66	.61	−	−	−	0.94	0.63–1.43	.77
Dyslipidemia	0.57	0.39–0.84	<.05	−	−	−	0.86	0.57–1.31	.47
Diabetes mellitus	1.18	0.83–1.68	.35	−	−	−	1.30	0.89–1.89	.18
Smoking	0.49	0.34–0.72	<.05	−	−	−	1.21	0.78–1.84	.40
eGFR<60 mL/min/1.73mL^2^	3.95	2.75–5.74	<.05	−	−	−	2.27	1.53–3.39	<.05
Killip class ≥2	3.56	2.36–5.24	<.05	−	−	−	2.44	1.52–3.83	<.05
Log peak CPK	0.74	0.49–1.12	.15	−	−	−	0.71	0.46–1.01	.71
LVEF <40%	2.39	1.56–3.55	<.05	−	−	−	1.78	1.12–2.74	<.05
Final TIMI flow ≤1	2.01	0.98–3.64	.05	−	−	−	1.83	0.81–3.56	.14
MACCE									
CAVB	1.33	0.77–2.16	.29	1.22	0.70–1.98	.46	0.91	0.50–1.55	.75
Age	1.04	1.03–1.06	<.05	1.04	1.03–1.06	<.05	1.04	1.02–1.06	<.05
Male gender	0.61	0.42–0.91	<.05	0.85	0.57–1.29	.44	0.77	0.60–1.34	.25
Hypertension	1.45	0.96–2.24	.08	−	−	−	1.26	0.83–1.98	.28
Dyslipidemia	0.81	0.55–1.26	.34	−	−	−	0.99	0.65–1.57	.98
Diabetes mellitus	1.41	0.99–2.01	.05	−	−	−	1.48	1.02–2.16	<.05
Smoking	0.57	0.39–0.81	<.05	−	−	−	1.06	0.69–1.62	.79
eGFR<60 mL/min/1.73mL^2^	2.82	1.98–4.03	<.05	−	−	−	1.79	1.21–2.65	<.05
Killip class ≥2	3.34	2.21–4.99	<.05	−	−	−	2.03	1.25–3.20	<.05
Log peak CPK	1.16	0.76–1.82	.50	−	−	−	1.10	0.70–1.73	.67
LVEF <40%	2.52	1.62–3.78	<.05	−	−	−	1.81	1.12–2.85	<.05
Final TIMI flow ≤1	2.04	1.00–3.70	<.05	−	−	−	1.87	0.83–3.66	.12

*Note:* Abbreviations as in Tables 1, 2, 3.

## 4 DISCUSSION

### 4.1 Major findings

In the present study, we demonstrated that approximately 6.3% of STEMI patients had a CAVB and its prevalence was more frequent in nonanterior STEMI patients than in anterior STEMI patients. Anterior STEMI patients with CAVB had worse outcomes compared to those without CAVB. Nonanterior STEMI patients with CAVB had equivocal short‐term outcomes compared to those without CAVB, whereas they had more all‐cause deaths compared to those without CAVB. Multivariate analysis demonstrated that CAVB was an independent predictor for all‐cause death and MACCE in anterior STEMI patients, whereas it was not an independent predictor in nonanterior STEMI patients. These findings indicate that CAVB in anterior STEMI patients, but not in nonanterior STEMI patients, is associated with worse outcomes, despite numerous technical advancements regarding early diagnosis and reperfusion therapy in the era of primary PCI.

### 4.2 Incidence of CAVB in STEMI patients

CAVB is one of the most common brady‐arrhythmias in STEMI patients, especially in inferior STEMI patients. The definition of AVB are different and it is not unified in many articles. For example, AVB was defined as “advanced” or “high‐degree” AVB in some studies,^5^, ^10^, ^11^, ^12^, ^13^, ^16^, ^17^, ^18^, ^19^, ^20^ while only CAVB was reported in others.^1^, ^2^, ^3^, ^4^, ^6^, ^7^, ^8^, ^9^, ^21^, ^22^ Moreover, monitoring timing was different whether it was recorded at admission or during hospitalization. Therefore, the frequency of AVB might be different among articles. In the present study, we focused on CAVB and showed that the incidence of CAVB was 6.3% in overall STEMI patients. This value was relatively higher compared with previous studies.^1^, ^2^, ^3^, ^4^, ^5^, ^6^, ^7^, ^8^ For example, Nguyen et al. reported that the incidence rate of CAVB in AMI patients was 5% to 7% between 1975 and 1990 and declined over time to 2% to 3% between 2000 and 2005.^1^ Aplin et al. also reported that among 6657 AMI patients, 340 (5.1%) developed CAVB between May 1990 and June 1992.^2^ It should be noted that their studies included both STEMI and nonSTEMI (NSTEMI) patients. Pokorney et al. recently reported that the incidence of high‐degree AVB (Mobitz type II or CAVB) was 0.4% (112/29677) in NSTEMI patients. This indicates that CAVB was infrequent complication in NSTEMI patients.^23^ Our study included only STEMI patients, and therefore, we may have found a relatively higher incidence of CAVB in the present study.

### 4.3 Impact of CAVB on clinical outcomes in STEMI patients

The association between CAVB and worse outcomes has been reported in several studies.^1^, ^2^, ^3^, ^4^, ^21^, ^22^ Nguyen et al. showed that AMI patients with CAVB had a higher hospital mortality than those without CAVB (43% vs 13%).^1^ Harpaz et al. further showed that AMI patients with CAVB had a higher 30‐day (21% vs 6%) and 1‐year mortality (35% vs 15%) than those without CAVB in the era of thrombolytic therapy.^3^ Spencer et al. showed that AMI patients with CAVB experienced significantly higher hospital death rates than those without CAVB (47% vs 15%).^4^ Furthermore, they showed that among discharged patients, 5‐year survival rate for patients with an anterior AMI with CAVB was lower than those without CAVB (37% vs 62%), whereas it was approximately 70% in patients with inferior AMI with CAVB. Consistent with these previous reports, our study in the era of primary PCI revealed that CAVB in nonanterior STEMI patients was also not independently associated with short‐term, nor long‐term mortality.

In contrast, anterior STEMI patients with CAVB had a higher mortality even in the era of primary PCI. Hreybe et al. previously showed that patients with anterior or lateral AMI were more likely to die prior to hospital discharge compared to those with an inferior or posterior AMI (11% vs 8%).^7^ In the present study, the mortality among the CAVB group was substantially higher in anterior STEMI than in nonanterior STEMI (82% vs 30%, *p* < .05). In anterior STEMI, CAVB patients died four times more than those without CAVB within the follow‐up period (82% vs 20%). This finding corresponds to the previous studies demonstrating that the negative prognostic impact of high‐degree AVB differs according to the location of infarct area, although much clearer in anterior STEMI.^4^, ^5^, ^6^, ^7^, ^12^, ^16^, ^22^ Furthermore, we found an association between anterior STEMI complicating CAVB and poor prognosis at 30‐day follow‐up (46% vs 7%, *p* < .05). In contrast, nonanterior STEMI patients with CAVB treated with primary PCI had a similar in‐hospital and 30‐day mortality compared with nonanterior STEMI patients without CAVB.

In the present study, time from onset to reperfusion was shorter in STEMI patients with CAVB compared with those without CAVB. AMI patients with CAVB may have additional symptoms such as dizziness or syncope primarily due to bradycardia, and they will be often referred to hospital immediately because of their poor conditions. This may be supported by our data showing that direct transfer to emergency room by the emergency services without going through primary care physicians was significantly more frequent in STEMI patients with CAVB than in those without.

### 4.4 Possible mechanism of CAVB in STEMI Patients

CAVB can occur in STEMI patients with either inferior or anterior infarction, though more common in inferior STEMI patients. The mechanism of conduction disturbance in STEMI patients is different according to the location of infarction. As expected, CAVB was more frequent in nonanterior STEMI. This is due to hypoperfusion of the AV nodal artery that is mainly supplied by RCA and rarely from LCX.^5^, ^24^, ^25^, ^26^, ^27^ This is also due to occlusion of RCA that secondarily increases acetylcholine release from the inferoposterior myocardium leading to an increasing parasympathetic tonus.^24^ Otherwise, CAVB is thought to be provoked by local release of potassium, adenosine, or a mixture of all the mentioned mechanisms.^5^

The conduction tissue of the AV node is usually resistant to permanent damage from ischemia due to the high intracellular contents of glycogen, the complex arterial blood supply such as from the septal perforators of LAD, and the capability of nutrient and oxygen absorption supplied from adjacent venous sinusoids.^28^, ^29^, ^30^ Narrow QRS complex junctional escape rhythms with a rate exceeding 40 bpm occur commonly, and pacing is not generally necessary in inferior STEMI patients because it is often transient. Meanwhile, in anterior STEMI patients, CAVB can occur suddenly and typically have unstable escape rhythms with wide QRS complexes and rates below 40 bpm (ventricular asystole may occur quite suddenly).^31^ CAVB generally develops as a result of extensive septal necrosis involving the His bundle or bundle branches traveling within the interventricular septum mainly supplied by LAD.^32^, ^33^ LAD also supplies the distal bundle branches and anterior STEMI must be very extensive to cause ischemia or necrosis of all these bundle branches. Although anterior STEMI causing CAVB rarely occur, extensive damage to the His bundle and the conduction system below the level of the His bundle caused by occlusion of LAD may explain the worse outcomes.^33^ Consistent with the previous studies showing the close association between CAVB and worse outcomes,^3^, ^10^, ^12^, ^16^, ^17^, ^18^, ^21^ anterior STEMI patients with CAVB had lower LVEF and higher peak‐CPK levels supporting extensive infarct size in the present study. Indeed, they experienced a higher incidence of cardiogenic shock and decompensated heart failure during hospitalization. Taken together, extensive conduction disturbance and myocardial damage in anterior STEMI patients with CAVB may be related to the worse outcomes.

### 4.5 Study limitations

Our study has several limitations. Firstly, we designed and performed this study in a single center. Secondly, we were unable to determine the precise onset or duration of CAVB and recovery timing from CAVB due to the retrospective nature of the study. Thus, we could not distinguish effects attributable to transient versus sustained CAVB during hospitalization. Thirdly, since CAVB was diagnosed based on ECG reports after contact of the patients with the emergency medical services, we may have missed transient CAVB before their contact. This may lead to an underestimate of the incidence of CAVB. Fourthly, ECG was not continuously monitored during all periods of hospitalization. Therefore, we might not have captured all CAVB episodes particularly in patients with asymptomatic transient CAVB. Furthermore, information on bundle branch or bifascicular block, and the duration of CAVB was lacking. Finally, because of consciousness disorder or severe condition in some patients, we could not interview all patients about previous medical therapy including beta‐blocker that was associated with development of bradycardia.

## 5 CONCLUSION

Although CAVB is a rare complication in anterior STEMI patients, it remains a poor prognostic complication even in the primary PCI era. Therefore, anterior STEMI patients with CAVB may require a more careful monitoring. Further studies are needed to determine the impact of CAVB and optimal treatment in these patients.

## Supporting information


**Appendix S1**: Supporting Information

## Data Availability

The data that support the findings of this study are available on reasonable request from the corresponding author. The data are not publicly available due to privacy or ethical restrictions.
