# Supplementary material for: Clinical impact of complete atrioventricular block in patients with ST‐segment elevation myocardial infarction
Source: Clin Cardiol. 2020 Nov 12;44(1):91–9. doi: 10.1002/clc.23510 (PMC7803372; doi:10.1002/clc.23510)
Supplement: Supplementary file 1 — Appendix S1: Supporting Information [file CLC-44-91-s001.docx]

**Supplemental materials**

**Clinical impact of complete atrioventricular block in patients with ST-segment elevation myocardial infarction**

Yosuke Kawamura MD, Hiroaki Yokoyama MD, PhD, Kazutaka Kitayama MD, Naotake Miura MD, Misato Hamadate MD, Daiki Nagawa MD, Masashi Nozaka MD, Masamichi Nakata MD, Fumie Nishizaki MD, PhD, Kenji Hanada MD, PhD, Takashi Yokota MD, PhD, Masahiro Yamada MD, PhD, Takumi Higuma MD, PhD, Hirofumi Tomita MD, PhD

**Supplementary Table.** Clinical data of anterior STEMI patients complicated with CAVB

| No. | Age | Gender | CAVB onset | No. of  diseased vessels | Final  TIMI | Time to reperfusion (min) | Killip | LVEF  (%) | Peak CPK  (IU/L) | Temporary pacing | Cardiogenic shock | Mortality | Cardiac death | Survival days | Device implantation |
| --- | --- | --- | --- | --- | --- | --- | --- | --- | --- | --- | --- | --- | --- | --- | --- |
| 1 | 77 | Male | after PCI | DVD | 3 | 600 | 1 | 18 | 5,378 | No | No | D | Yes | 348 | No |
| 2 | 83 | Female | after PCI | DVD | 2 | 477 | 1 | 19 | 14,286 | Yes | No | D | Yes | 1 | No |
| 3 | 87 | Male | after PCI | TVD | 3 | 152 | 4 | 49 | 8,964 | Yes | Yes | D | No | 832 | Yes |
| 4 | 68 | Female | after PCI | DVD | 2 | 310 | 4 | 21 | 13,502 | No | Yes | D | Yes | 16 | No |
| 5 | 70 | Male | after PCI | DVD | 3 | 344 | 2 | 45 | 23,990 | No | Yes | D | Yes | 309 | No |
| 6 | 87 | Male | before PCI | DVD | 2 | 150 | 4 | 38 | 7,197 | Yes | Yes | D | Yes | 1 | No |
| 7 | 77 | Male | during PCI | DVD | 2 | 494 | 3 | 30 | 12,259 | Yes | No | D | Yes | 128 | No |
| 8 | 43 | Male | after PCI | SVD | 2 | 103 | 1 | 53 | 9,345 | No | No | A | No | 1,191 | No |
| 9 | 55 | Male | before PCI | TVD | 2 | 161 | 4 | 40 | 23,439 | Yes | Yes | D | Yes | 3 | No |
| 10 | 77 | Male | after PCI | SVD | 2 | 568 | 1 | 40 | 12,009 | No | Yes | D | Yes | 27 | No |
| 11 | 81 | Female | before PCI | SVD | 2 | 282 | 4 | 30 | 2,094 | Yes | Yes | A | No | 320 | No |

STEMI indicates ST-segment elevation myocardial infarction, CAVB; complete atrioventricular block, PCI; percutaneous coronary intervention, SVD; single-vessel disease, DVD; double-vessel disease, TVD; triple-vessel disease, TIMI; Thrombolysis in Myocardial Infarction, LVEF; left ventricular ejection fraction, CPK; creatine phosphokinase, D; dead, A; alive.

**Supplementary Figure.** Flow chart of the study. STEMI indicates ST-segment elevation acute myocardial infarction, CAVB; complete atrioventricular block, PCI; percutaneous coronary intervention.
